# Benchmarking short-term postoperative mortality across neurosurgery units: is hospital administrative data good enough for risk-adjustment?

**DOI:** 10.1007/s00701-023-05623-5

**Published:** 2023-05-27

**Authors:** Adam J Wahba, Nick Phillips, Ryan K Mathew, Peter J Hutchinson, Adel Helmy, David A Cromwell

**Affiliations:** 1 Clinical Effectiveness Unit, Royal College of Surgeons of England, 35-43 Lincoln’s Inn Fields, London, WC2A 3PE UK; 2 Leeds Institute of Medical Research, School of Medicine, Worsley Building, University of Leeds, Leeds, LS2 9JT UK; 3 Department of Neurosurgery, Leeds Teaching Hospitals NHS Trust, Great George Street, Leeds, LS1 3EX UK; 4 Department of Research, Royal College of Surgeons of England, 35-43 Lincoln’s Inn Fields, London, WC2A 3PE UK; 5 Division of Neurosurgery, Addenbrooke’s Hospital, Cambridge University Hospitals NHS Foundation Trust, Hills Road, Cambridge, CB2 0QQ UK; 6 Department of Health Services Research & Policy, London School of Hygiene & Tropical Medicine, 15-17 Tavistock Place, London, WC1H 9SH UK

**Keywords:** Risk-adjustment, Mortality, Neurosurgery, Neurovascular, Neuro-oncology, Hospital administrative data

## Abstract

**Background:**

Surgical mortality indicators should be risk-adjusted when evaluating the performance of organisations. This study evaluated the performance of risk-adjustment models that used English hospital administrative data for 30-day mortality after neurosurgery.

**Methods:**

This retrospective cohort study used Hospital Episode Statistics (HES) data from 1 April 2013 to 31 March 2018. Organisational-level 30-day mortality was calculated for selected subspecialties (neuro-oncology, neurovascular and trauma neurosurgery) and the overall cohort. Risk adjustment models were developed using multivariable logistic regression and incorporated various patient variables: age, sex, admission method, social deprivation, comorbidity and frailty indices. Performance was assessed in terms of discrimination and calibration.

**Results:**

The cohort included 49,044 patients. Overall, 30-day mortality rate was 4.9%, with unadjusted organisational rates ranging from 3.2 to 9.3%. The variables in the best performing models varied for the subspecialties; for trauma neurosurgery, a model that included deprivation and frailty had the best calibration, while for neuro-oncology a model with these variables plus comorbidity performed best. For neurovascular surgery, a simple model of age, sex and admission method performed best. Levels of discrimination varied for the subspecialties (range: 0.583 for trauma and 0.740 for neurovascular). The models were generally well calibrated. Application of the models to the organisation figures produced an average (median) absolute change in mortality of 0.33% (interquartile range (IQR) 0.15–0.72) for the overall cohort model. Median changes for the subspecialty models were 0.29% (neuro-oncology, IQR 0.15–0.42), 0.40% (neurovascular, IQR 0.24–0.78) and 0.49% (trauma neurosurgery, IQR 0.23–1.68).

**Conclusions:**

Reasonable risk-adjustment models for 30-day mortality after neurosurgery procedures were possible using variables from HES, although the models for trauma neurosurgery performed less well. Including a measure of frailty often improved model performance.

**Supplementary Information:**

The online version contains supplementary material available at 10.1007/s00701-023-05623-5.

## Introduction

Quality improvement (QI) programmes that investigate organisation-level outcomes should use risk-adjusted indicators to ensure the benchmarking of organisations is fair [4]. Risk-adjustment aims to remove the effect of differences in the distribution of patient characteristics across organisations in the outcome indicator values, without which assessments of performance might be inaccurate due to confounding. Effective risk adjustment models are therefore required to allow clinicians to have confidence in using the indicators for quality assurance and informing QI activities.

Various risk adjustment models have been used for neurosurgical outcome indicators [16, 20, 23, 31]. Ideally, these models are developed using patient variables from clinical datasets and incorporate attributes specific to the neurological condition. These may include the type of neurosurgical operation or indication for a procedure, and these clinical factors have been shown to be important components of risk-adjustment in neurosurgery [16, 23]. In other situations, the indicators are derived using administrative hospital datasets and the variables available are typically more generic, such as age, sex and a measure of comorbidity [20, 31]. Administrative hospital datasets (like the English Hospital Episode Statistics) have been used to produce effective risk adjustment models for short-term outcomes like 30-day post-operative mortality for various surgical procedures [5]. There is recent evidence supporting the use of administrative data to investigate comparative mortality rates in neurosurgery [35], but the performance of these type of risk adjustment models has not been evaluated for neurosurgical procedures. Such models are required for producing risk-adjusted organisation-level outcome indicators within the National Neurosurgical Audit Programme (NNAP) of the Society of British Neurological Surgeons (SBNS) [33].

The aim of this research was to assess the performance of risk-adjustment models for 30-day mortality after neurosurgical procedures when developed using hospital administrative data. The study examined the performance of models for an overall cohort of neurosurgical patients and for specific subspecialties: neuro-oncology, neurovascular and trauma neurosurgery.

## Methods

### Data source and cohort definition

The study used an extract of Hospital Episode Statistics (HES) data that covered neurosurgery activity in National Health Service (NHS) hospitals in England during the 5 years from 1 April 2013 to 31 March 2018. HES is the hospital administrative data for NHS funded hospital activity in England and contains data on patient demographics, diagnoses, procedures and administrative information. Records describe the care delivered under the care of a consultant and can capture data on up to 20 procedures (date of operation and type of procedure) and 24 medical conditions. Procedures are coded using the UK Office of Population Censuses and Surveys (OPCS, version 4) classification; medical conditions are coded using the International Classification of Diseases, version 10 (ICD-10).

The study cohort included adult patients (≥18 years) who were admitted to a neurosurgery unit, either electively or as an emergency/transfer and who underwent a neurosurgical procedure in one of three main subspecialties (neuro-oncology, neurovascular and trauma neurosurgery). After identification of records containing the relevant procedures, the primary diagnosis was used to exclude any patients that did not have pathology relevant to the subspecialty. (The various types of procedure, OPCS codes and ICD-10 codes are shown in Table S1, supplementary material). The primary outcome measure was all-cause 30-day post-operative mortality. The date of death was obtained from the Civil Registration of Mortality data and linked to the HES records [25].

### Variable definition

The study used variables available from the HES database, with values derived from the index admission. These included: patient demographics (age, sex and socioeconomic deprivation), method of admission (elective or emergency) and details of the neurosurgical conditions and comorbidities.

Area-level socioeconomic deprivation was measured using the Index for Multiple Deprivation (IMD), with the analysis grouping areas into quintiles based on the ranks of their overall IMD values. Frailty was measured using the secondary care administrative records frailty (SCARF) index [15]. The SCARF index is based on the ‘accumulation of deficits’ model of frailty. ICD-10 diagnosis codes are used to define 32 deficits that cover functional impairment, geriatric syndromes, problems with nutrition, cognition and mood and medical comorbidities. The index uses four categories (‘fit’, mild, moderate and severe frailty), with severe frailty defined as the presence of six or more deficits.

Comorbidity burden was measured in two ways, using (i) the Royal College of Surgeons of England (RCS) Charlson Comorbidity Index (CCI) and (ii) the Elixhauser Score (ES) (van Walraven modification) [2, 34]. The CCI was included in the models as a simple count of comorbidities (0, 1, 2, 3+). The ES was included as a categorical variable, with the weighted scores of the ES divided into six patient groups of reasonable size (−11 to −1, 0, 1 to 4, 5 to 8, 9 to 12, 13+). The CCI and ES were implemented with a one-year ‘look-back’ period, which increases the ability of the measures to capture chronic conditions and allows them to distinguish comorbidity from acute illness for several diagnoses in the index admission (for example, myocardial infarction is only flagged from previous admissions and not the index admission). (See Tables S2 and S3 in supplementary material).

### Risk-adjustment models and statistical analysis

The study was performed as a complete case analysis. Any records that had missing data in the explanatory variables were excluded. Descriptive statistics were used to summarise the characteristics of patients undergoing the various procedures. The relationship between postoperative mortality and patient characteristics was initially explored using bivariate analyses (Wilcoxon rank-sum tests for medians and *Χ*^2^ tests for proportions). Then, multivariable logistic regression models were used to investigate the relationships between postoperative mortality and all potential variables. To account for potential clustering effects within NHS trusts, robust standard errors were estimated using the Huber-White sandwich method.

The process of model development proceeded in a series of stages. First, the performance of a basic model (which included the variables age (years), sex (male / female), subspeciality and method of admission (elective/emergency) was evaluated using the data for the whole cohort. The relationship of age with 30-day mortality was assessed to see whether it was linear or non-linear using a range of transformations including fitting fractional polynomials [29]. Age was ultimately included as a continuous variable without any transformation (i.e. it had a linear relationship with 30-day post-operative mortality). Extreme values of age were winsorized to avoid them having excessive influence; that is, age was restricted to lie between 45 and 90. The final step was to test for interactions between the model variables as it was hypothesised that risk factors might interact to increase the risk of death above the combined individual effect of each variable.

Second, the impact on performance of adding other variables to the basic model was evaluated. The variables were added in the following sequence: indices of socioeconomic deprivation, frailty and comorbidity using either CCI or ES (i.e. Basic + IMD + SCARF + CCI, then Basic + IMD + SCARF + ES).

Third, the process was repeated for the three neurosurgical subspecialties (with the omission of the subspecialty variable from the basic model). The performance of the final models for each subspecialty was compared to the performance of the risk-adjustment model produced for the overall cohort to assess whether a single model was sufficiently versatile to work across neurosurgery. The performance of each subspecialty model was also evaluated on a key procedure to check its performance was retained for individual subgroups (resection of intracerebral tumour, clipping of aneurysm and evacuation of acute subdural haematoma).

The BIC (Bayesian information criterion) was used to assess the quality of each model, and the relative degree of improvement produced by adding each variable. The BIC is a method of evaluating the performance of regression models with different sets of variables. The BIC decreases in value for models that fit the data better but it includes a penalty that increases the BIC value as more variables are added to a model, to prevent overfitting. It therefore allows the performance of different models to be compared, with the best performance corresponding to the minimum BIC value. The predictive performance of each logistic regression model was evaluating by measuring its discrimination and calibration. Discrimination measures the ability of the model to distinguish between those who did and did not die within 30 days of surgery and is reported using the c-statistic (or area under the receiver operating characteristic (ROC) curve). C-statistic values typically fall between 0.5 (indicating the model is no better at predicting the outcome than a random guess) and 1.0 (perfect discrimination). Calibration measures the agreement between the observed mortality and expected mortality predicted by the model and is a measure of goodness of fit. This was evaluated graphically with calibration plots with patients grouped into 10 categories of increasing risk. Calibration was also evaluated using the Brier score which takes on a value between 0 and 1. It measures the accuracy of model predictions and lower scores indicate better calibration.

Finally, the distribution of predicted risk across the 24 NHS neurosurgical units in England was evaluated using the models that showed the best calibration. The differences in unadjusted and adjusted mortality rates for each unit were explored for the overall cohort and in each of the subspecialties.

Data analysis was performed using Stata, version 17 (StataCorp LP, College Station, TX).

## Results

From 1 April 2013 to 31 March 2018, 50,748 patients underwent a neurosurgical procedure within the selected subspecialties. Of these, 1704 (3.4%) had missing data and were excluded. The final study cohort included 49,044 patients. The characteristics of patients in the overall cohort and the three subspecialties are shown in Table 1. In the overall cohort, the proportions of male and females were equal, but trauma patients were predominantly male (72.6%) and neurovascular patients were predominantly female (male=35.9%). The proportion of patients scored as ‘fit’ on the frailty index was 31.1% overall. However, it varied in the subspecialty groups being low among trauma patients (15.1%) but high among neuro-oncology patients (40.4%). The basic pattern of 30-day postoperative mortality across the cohort is summarised in Table 2. The overall mortality rate was 4.9% and fell between 0.4 and 11.9% in the subspecialty groups stratified by admission method and sex.

**Table 1:** Characteristics of patients who had a neurosurgical procedure between April 2013 and March 2018 in English NHS hospitals for the overall cohort and the three subspecialties

**Overall cohort**			**Neurovascular surgery**		
No. of patients	49,044		No. of patients	14,695	
Average age, (median, IQR)	58 (46–69)		Average age, (median, IQR)	56 (47–66)	
Male, *n* (%)	24,703	50.4	Male, *n* (%)	5270	35.9
Emergency admission, *n* (%)	25,199	51.4	Emergency admission, *n* (%)	8666	59.0
Comorbidities, *n* (%)			Comorbidities, *n* (%)		
0	25,424	51.8	0	5929	40.3
1	13,604	27.7	1	5113	34.8
2	6780	13.8	2	2576	17.5
3+	3236	6.6	3+	1077	7.3
Frailty, *n* (%)			Frailty, *n* (%)		
Fit	15,250	31.1	Fit	3921	26.7
Mild	16,504	33.7	Mild	5322	36.2
Moderate	11,975	24.4	Moderate	4039	27.5
Severe	5315	10.8	Severe	1413	9.6
**Neuro-oncology surgery**			**Trauma neurosurgery**		
No. of patients	24,354		No. of patients	9995	
Average age, (median, IQR)	58 (46–67)		Average age, (median, IQR)	66 (45–79)	
Male, *n* (%)	12,175	50.0	Male, *n* (%)	7259	72.6
Emergency admission, *n* (%)	6539	26.9	Emergency admission, *n* (%)	9995	100
Comorbidities, *n* (%)			Comorbidities, *n* (%)		
0	13,884	57.0	0	5590	55.9
1	6290	25.8	1	2207	22.1
2	2932	12.0	2	1278	12.8
3+	1248	5.1	3+	920	9.2
Frailty, *n* (%)			Frailty, *n* (%)		
Fit	9829	40.4	Fit	1513	15.1
Mild	8310	34.1	Mild	2883	28.8
Moderate	4820	19.8	Moderate	3108	31.1
Severe	1395	5.7	Severe	2491	24.9

**Table 2:** 30-day postoperative mortality rates (%) for neurosurgical procedures (April 2013 – March 2018) for the overall cohort and subspecialties, stratified by method of admission and patient sex

	All Neurosurgery	Neuro-oncology surgery	Neurovascular surgery	Trauma neurosurgery
Overall mortality rate	4.9	2.8	4.0	11.3
Elective admissions	1.6	1.9	0.6	−
Male	1.9	2.3	0.4	−
Female	1.3	1.5	0.8	−
Emergency admissions	8.0	5.2	6.4	11.3
Male	8.3	5.2	5.3	11.0
Female	7.6	5.1	7.0	11.9

Regarding the development of the models, age was fitted as linear relationship with mortality in the overall basic model. None of the variable interactions explored had a significant impact on model performance so these were excluded from the final models. The adjusted odds ratios for each variable in the four models summarising the relationship between patient characteristics and mortality are shown in Table S4 (supplementary material).

The BIC values typically demonstrated improvement in the quality of the models with each iteration, but there were several exceptions. The addition of deprivation categories resulted in a negligible improvement to the overall model and it did not improve the quality of the trauma and neuro-oncology subspecialty models. The addition of a comorbidity index did not significantly improve the quality in the neuro-oncology model. The addition of variables to the basic (model 1) neurovascular model did not lead to any improvement. Overall, the pattern of changes in the BIC generally conformed to improvements in model calibration.

Predictive performance in terms of discrimination improved with the addition of variables to the overall cohort model (Table 3). The same pattern was observed in the subspecialty models, where model 5 had the highest discrimination. Discrimination was moderate for the neuro-oncology and neurovascular models at 0.735 and 0.740, respectively. It was poor in the trauma model at 0.583. The subspecialty models showed better discrimination than the overall model applied to the subspecialty groups. However, absolute increases in discriminatory ability were small, particularly in the overall cohort with an increase of just 0.007. In some instances, the models showed poor discrimination, but were still adequately calibrated. The c-statistic depends on the range of predictions and can be low if the range is small and not close to zero. For example, model 3 for trauma neurosurgery had a c-statistic of only 0.552 yet still had reasonable calibration.

**Table 3:** Discriminatory ability of risk-adjustment models that predict 30-day mortality for neurosurgical procedures

Procedure group	Model 1: basic – (age, sex, admission type)	Model 2: basic + deprivation	Model 3: basic + deprivation + frailty	Model 4: basic + deprivation + frailty + comorbidity (CCI)	Model 5: basic + deprivation + frailty + comorbidity (ES)	Difference between the lowest and highest value
Overall cohort model	0.729 (0.720–0.738)	0.728 (0.719–0.737)	0.733 (0.724–0.742)	0.733 (0.724–0.742)	0.735(0.726–0.745) *	0.007
Overall cohort model applied to subspecialties						
Neuro-oncology surgery	0.687 (0.668–0.707)	0.678 (0.658–0.698)	0.702(0.682–0.722) *	0.692 (0.672–0.713)	0.696 (0.677–0.716)	0.024
Neurovascular surgery	0.726 (0.709–0.743)	0.731(0.715–0.748) *	0.712 (0.695–0.729)	0.718 (0.701–0.735)	0.718 (0.701–0.735)	0.019
Trauma neurosurgery ^a^	0.477 (0.460–0.493)	0.483 (0.466–0.501)	0.516 (0.498–0.534)	0.522 (0.504–0.540)	0.544 (0.525–0.562) *	0.067
Subspecialty models						
Neuro-oncology surgery	0.694 (0.675–0.713)	0.695 (0.676–0.714)	0.728 (0.709–0.747)	0.734 (0.716–0.752)	0.735 (0.716–0.753) *	0.041
Neurovascular surgery	0.729 (0.712–0.745)	0.733 (0.716–0.750)	0.734 (0.718–0.750)	0.739 (0.723–0.755)	0.740(0.724–0.756) *	0.011
Trauma neurosurgery ^a^	0.528 (0.511–0.546)	0.539 (0.522–0.557)	0.552 (0.534–0.570)	0.558 (0.541–0.575)	0.583 (0.565–0.600) *	0.055

C-statistics are derived from the area under the ROC curve and the 95% CI is shown in parentheses

^*^Model with the highest c-statistic for each group

^a^Admission type does not apply — all procedures were emergency admissions

**Fig. 1:**
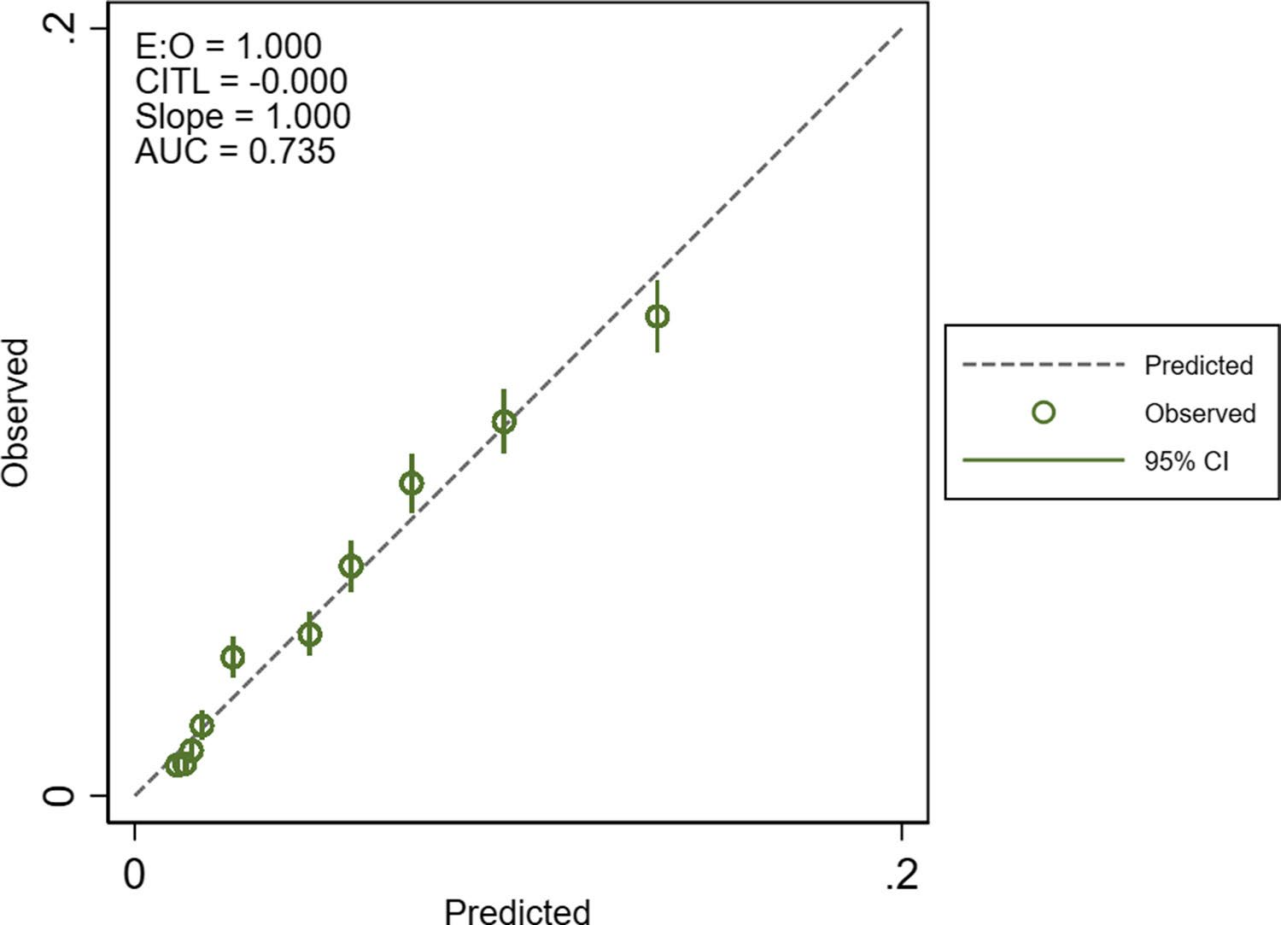
Calibration plot for the overall cohort model. Model 5 (basic + deprivation + frailty + comorbidity (ES)) was most well calibrated of the model iterations. E:O – calibration intercept, CITL – calibration in-the-large, AUC – area under the receiver operating characteristic curve (c-statistic)

**Fig. 2:**
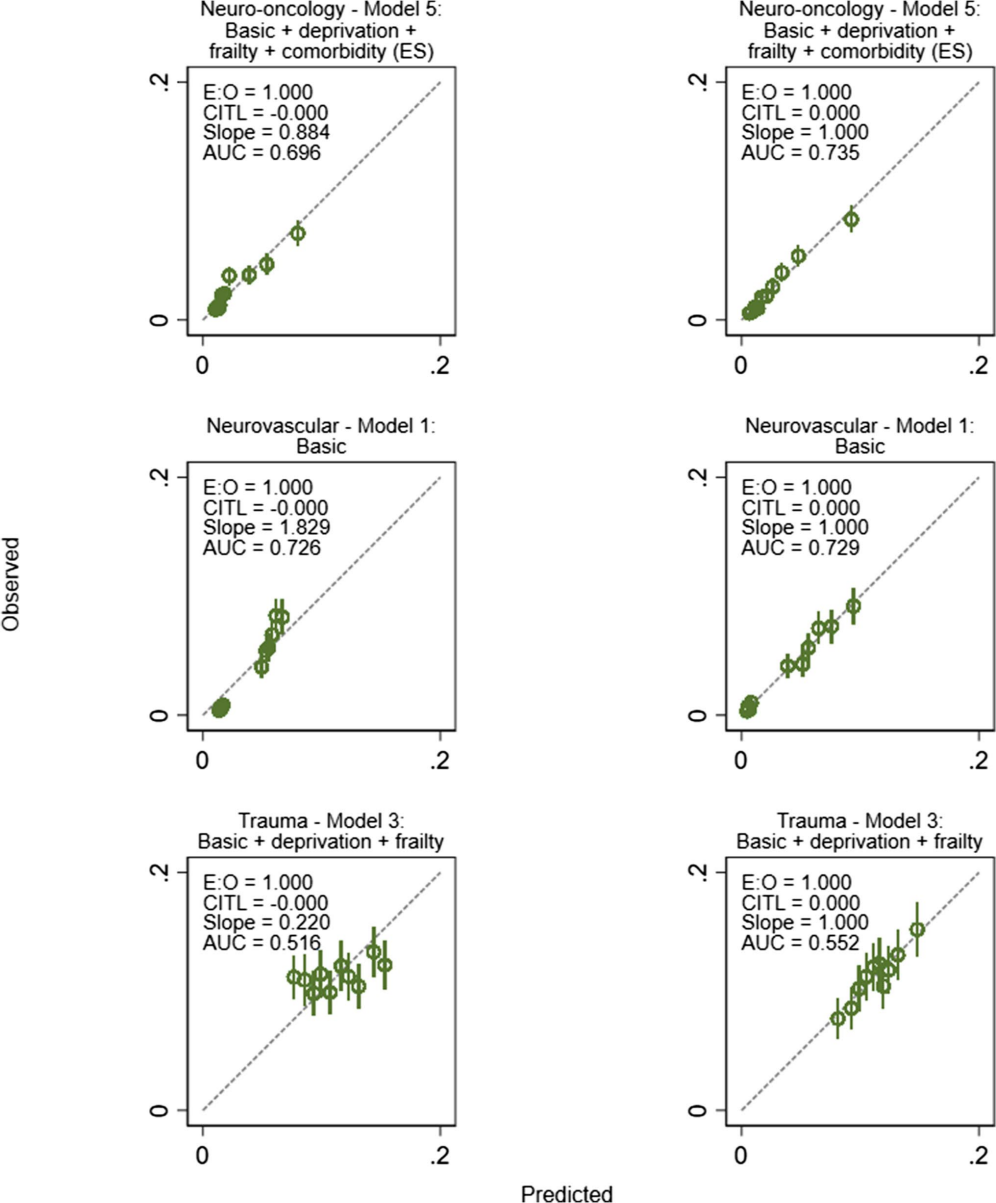
Calibration plots comparing the performance of the overall cohort model applied to subspecialties (left column) and the subspecialty models (right column). Model 5 was most well calibrated for neuro-oncology surgery, model 1 for neurovascular surgery and model 3 for trauma neurosurgery. The subspecialty models were better calibrated than the overall model across the subspecailties. E:O – calibration intercept, CITL – calibration in-the-large, AUC – area under the receiver operating characteristic curve (c-statistic)

Figure 1 shows that the overall cohort model was well calibrated, with model 5 showing the closest agreement between observed and predicted mortality rates across the ten risk groups. Figure 2 shows that the subspecialty models were better calibrated than the overall model applied to the subspecialty groups, with good agreement between observed and predicted mortality. The changes in calibration with the addition of variables was different across the study groups (Figure S1 shows the progressive change for models 1–5). It tended to improve with the addition of variables to the overall cohort model, it made only a slight difference to the neuro-oncology model, and it worsened the neurovascular model where model 1 was best. The additional of frailty to the trauma model improved calibration but not the addition of comorbidity. The subspecialty models retained their performance when tested in a key procedure within each group, with similar c-statistics (Table S6) and good calibration (Figure S2). The Brier scores for the overall cohort models (1 -5) were 0.045. The scores in the subspecialty models were 0.099 – 0.100 for trauma neurosurgery, 0.026 – 0.027 for neuro-oncology surgery and 0.038 for neurovascular surgery.

**Fig. 3:**
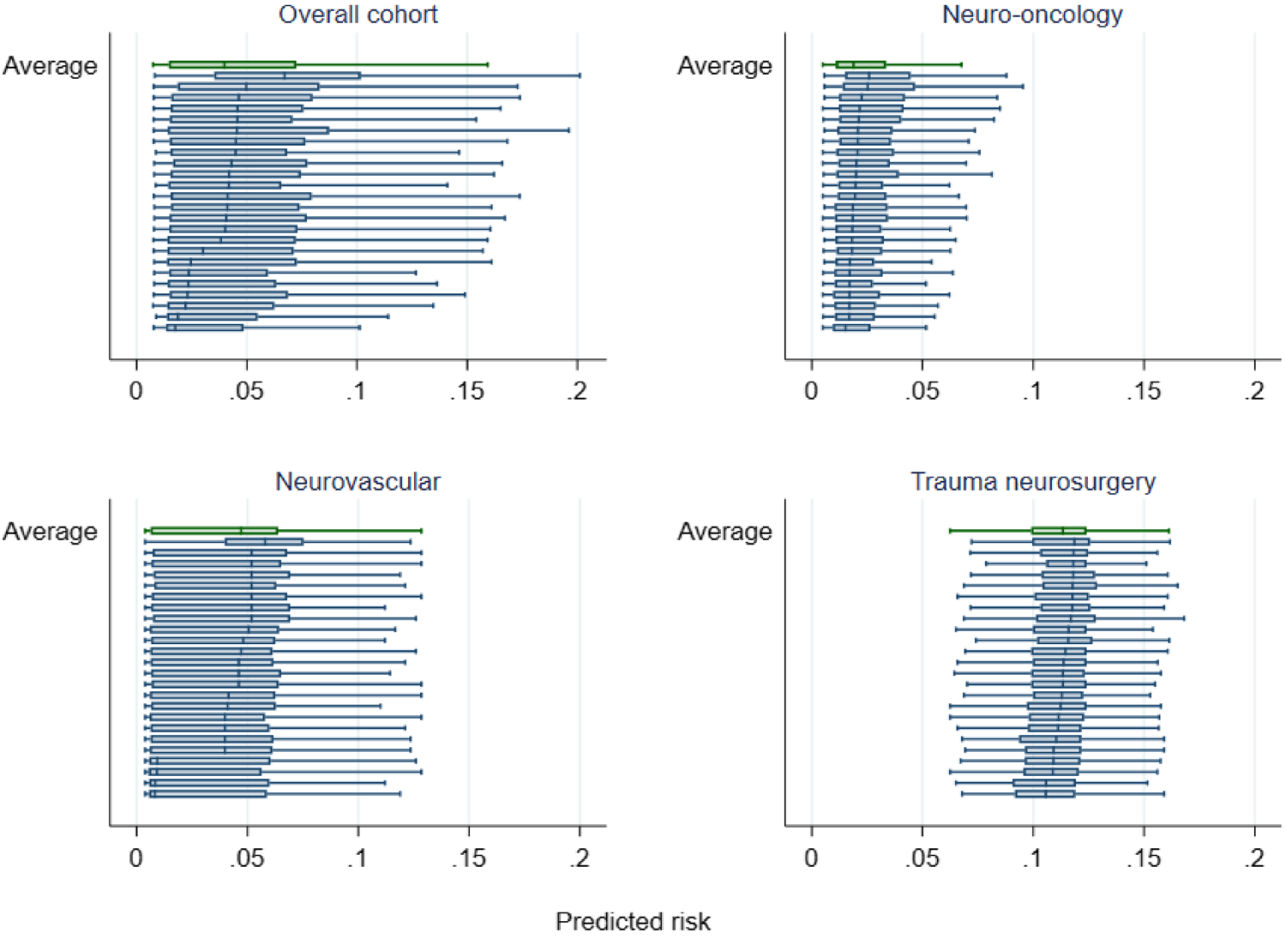
Box plots showing the distribution of predicted risk in each of the neurosurgical procedure groups across the 24 neurosurgical units in England. The risk predictions were generated using the best performing overall model and subspecialty models. Note: The middle line represents the median, the box represents the 25th and 75th centiles and the capped bars represent the lower and upper adjacent values

**Fig. 4:**
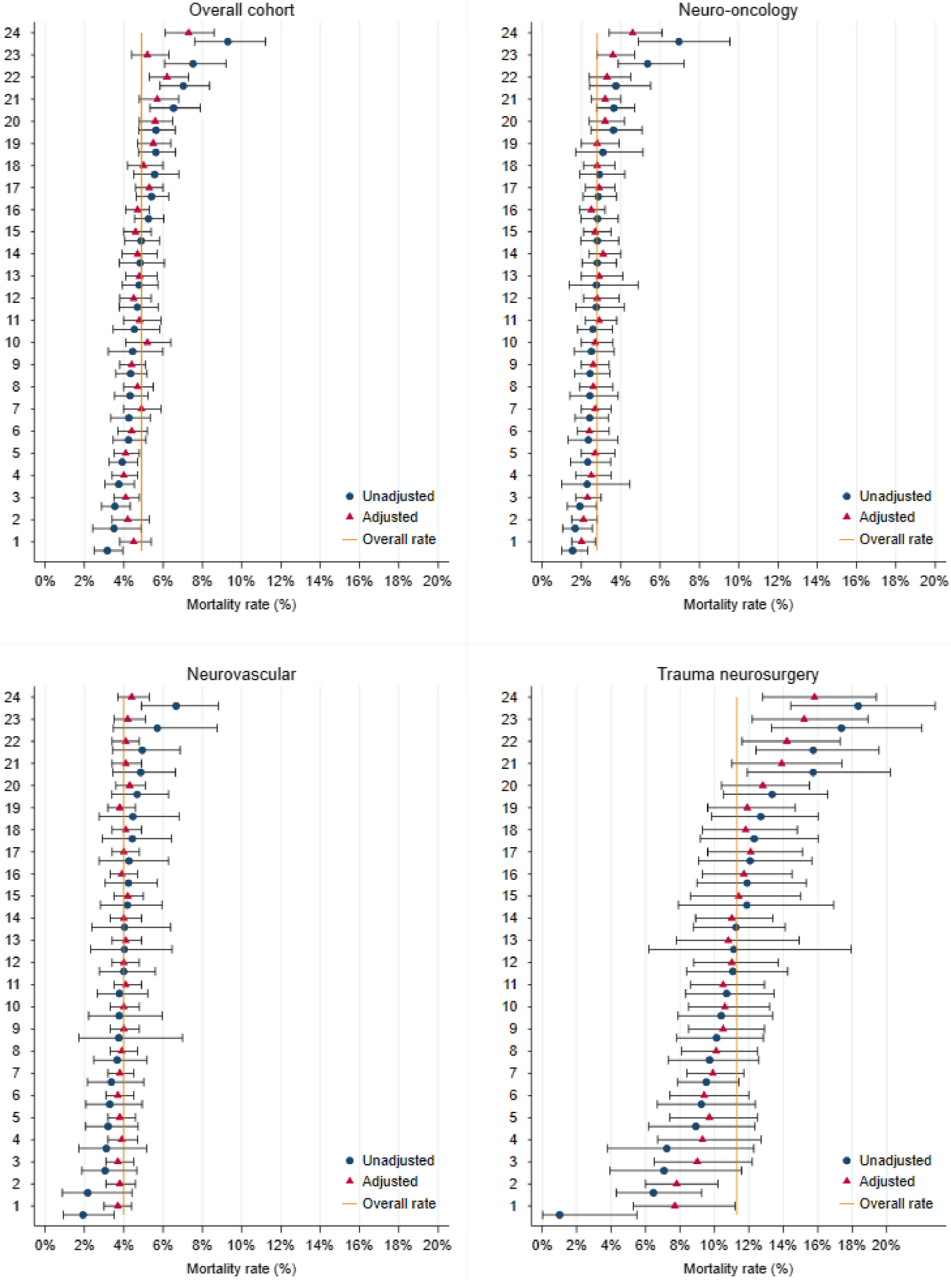
Caterpillar plots of unadjusted and risk-adjusted mortality rates for the 24 neurosurgical unit in England. Rates were adjusted using the best performing overall model and subspecialty models

The distribution of predicted risk varied across neurosurgical units and the extent of variation differed by procedure type (Fig. 3). Variation was most pronounced in neurovascular surgery and more limited in trauma. The range of organisational mortality rates for the overall cohort was 3.2 to 9.3%. Application of the models to the organisation figures produced an average (median) absolute change of 0.33% (interquartile range (IQR) 0.15–0.72) for the overall cohort model. Median changes for the subspecialty models were 0.29% (neuro-oncology, IQR 0.15–0.42), 0.40% (neurovascular, IQR 0.24–0.78) and 0.49% (trauma neurosurgery, IQR 0.23–1.68). Figure 4 shows the unadjusted and adjusted 30-day mortality rates for each of the 24 neurosurgical units; risk-adjustment tended to pull mortality rates towards the average, particularly at the higher and lower mortality rates.

## Discussion

### Interpreting model performance

The objective of this study was to assess the performance of risk adjustment models based on routinely collected national data that predict 30-day post-operative mortality, identifying which variables were important for risk-adjustment, and determining if a single model was sufficiently versatile to work across neurosurgery or if subspecialty models performed better.

Model 5 performed best in the overall cohort; it had moderate discrimination and was well calibrated. A model which works well across a broad range of procedures is an important tool for quality improvement; neurosurgical quality improvement programmes often pool procedures for analysis to evaluate quality across the breadth of neurosurgical practice [3, 27, 33]. The overall model performed less well when applied to the subspecialties than the models developed directly in the subspecialty groups. The subspecialty models were well calibrated, with good agreement between observed and predicted mortality. The discriminative ability of the models varied, with c-statistics that were poor (trauma) or moderate (neuro-oncology and neurovascular). However, a well calibrated model may have a poor c-statistic when the predicted risks are not close to either 0 or 1 (as with the trauma model) and a perfectly calibrated model may only be able to achieve a c-statistic well below 1 [9]. While discrimination is an important measure of model performance, calibration is often considered more important in the context of risk-adjustment [1, 22]. The overall and subspecialty models reduced between-organisation variation in mortality rates.

Interestingly, for neurovascular surgery the simplest model showed the best performance. The addition of deprivation, frailty and comorbidity indices impaired calibration; the more complex models were worse at predicting the number of deaths than the model that simply used age, sex and admission type. There may be several reasons for this. The risk factors for early mortality in neurovascular surgery are mainly related to disease severity or characteristics (for example, severity of subarachnoid haemorrhage (SAH) or size and location of aneurysm) [14]. There is also good evidence that age has a significant impact on poor outcomes in aneurysmal SAH [10]. Some causes of early mortality — which include poor grade SAH, or serious peri-operative events such as stroke or aneurysm re-rupture – may not be much affected by patients’ general health status. As such, age and the method of admission were strong predictors of post-operative mortality, while the effects of comorbidity and frailty are less important [18].

The neurovascular surgery model was well calibrated and had moderate discrimination despite the absence of markers of disease severity or clinical features of vascular lesions. It performed similarly well to a prediction model for SAH developed using observational data from several countries, which included age, premorbid hypertension and several clinical components including a severity score and imaging findings [14]. The corollary is that routinely collected data could be used for monitoring of provider performance in neurovascular surgery, particularly in the absence of national registry data. An optimal model may incorporate data from both HES and pathology-specific predictors.

Traumatic brain injury (TBI) is a heterogeneous disease process and outcomes are multifactorial [8]. The relative underperformance of the models in trauma is likely to arise from the lack of several important prognostic markers for TBI such as Glasgow Coma Scale and pupil reactivity, which are not recorded in HES [24]. Data from the Trauma Audit and Research Network (TARN) has been used to evaluate organisational-level outcomes and these studies have used a risk-adjustment model that includes important TBI risk factors [6, 21].

Across the models, there was little difference in performance between models 4 and 5. The models using ES had equal or higher discrimination than CCI, but the choice of comorbidity index made little difference to model calibration. Some evidence suggests that the ES may be a superior predictor to the CCI, but in our study the observed differences in performance were small [13, 32]. In fact, the addition of a measure of comorbidity had far less impact than the frailty index, particularly to the overall cohort and trauma models, suggesting that frailty was a more important factor to risk adjustment.

### The importance of frailty in neurosurgery

Frailty is a state of poor physiological reserve and increased vulnerability to stressors [12]. It is increasingly recognised as an important consideration in neurosurgical decision making and measuring outcomes [10]. A recent systematic review reported that there are only a small number of studies about the effect of frailty on neurosurgical outcomes but it showed that frailty is a significant and independent risk factor for neurosurgical outcomes [26]. More recently, two large retrospective cohort studies demonstrated that increasing frailty was associated with worse outcomes following brain tumour resections and cranial neurosurgery in general [17, 30]. Data from the CENTRE-TBI study also showed that greater frailty was significantly associated with worse outcomes after TBI [11].

The inclusion of the SCARF index often improved model performance and its use for risk-adjustment in neurosurgery is a novel aspect of this study. There is a degree of overlap between the frailty and comorbidity indices because the SCARF index measures several medical conditions that are in the comorbidity indices; it may be that those comorbidities which introduced risk of a worse outcome were already accounted for by the frailty index. Alternatively, a systematic review suggested that prediction models which measured physical status were superior to comorbidity indices in predicting morbidity in patients undergoing elective intracranial tumour resection [28].

### Risk-adjustment for performance monitoring

The distributions of patients’ predicted risk were similar across the 24 neurosurgical units in England and between the different study groups. This could arise because neurosurgical units generally operate on similar patients. Or it could be because the models capture only a limited number of characteristics (omitting variables such as tumour type or location) [31].

The effect of risk-adjustment on mortality rates was to pull rates towards the national average. The extent of adjustment underlines the need for comparative outcomes to be risk-adjusted, and, in the context of performance monitoring, suggests that adequate risk adjustment may reduce the risk of false alerts for comparatively high mortality rates.

The methodological approach adopted in this study can be readily transferred to other healthcare systems. Many administrative datasets and registries use ICD-10 or equivalent coding systems for recording conditions, and datasets will generally include most of the variables used in the models. The comorbidity indices evaluated here have been used widely in registry and administrative data research [20]. Similarly, the SCARF frailty index, or suitable alternative frailty indices, can be derived using ICD-10 diagnosis codes from registry or administrative data for risk-adjusted performance measures [19, 36].

### Limitations

The study was a complete case analysis with 3.4% of records excluded. This could have introduced selection bias because the nature of the missing data is unknown, but the proportion of records excluded was small and these records were not concentrated in any neurosurgical units. Administrative data are subject to errors in the accuracy and completeness of the clinical coding of diagnoses and procedures. The quality of HES data has improved over time but comorbidity may be under-recorded in hospital administrative data [7, 32]. Furthermore, the look-back method used to distinguish comorbidity from acute illness could introduce an unknown level of bias for those patients who did not have a previous admission in the look-back period. Missing and inaccurate data may cause risk-adjustment models to under-perform and so not adjust outcome measures sufficiently for the fair comparison of providers. While administrative datasets are widely used for performance monitoring, this remains a significant concern about the reliability of quality assurance programmes that rely on these data.

## Conclusions

This study produced risk-adjustment models for 30-day mortality after neuro-oncology and neurovascular procedures using HES data that had moderate discrimination and were well calibrated. The inclusion of a frailty index often improved model performance; frailty may have an important influence on early mortality following neurosurgical procedures. The distribution of predicted risk and extent of adjustment varied across the 24 neurosurgical units in England, which supports the necessity for quality indicators to be risk-adjusted. Further work should explore how the models could be refined for specific neurosurgical subspecialties, including where possible proxy measures for disease severity or procedure-related risk factors.

## Supplementary information

ESM 1 (DOCX 214 kb)

## Data Availability

No additional data available.

## Abbreviations

BIC: Bayesian information criterion
CCI: Charlson Comorbidity Index
ES: Elixhauser Score
HES: Hospital Episode Statistics
ICD: International Classification of Diseases
IMD: Index of Multiple Deprivation
NHS: National Health Service
NNAP: National Neurosurgical Audit Programme
OPCS: Office of Population Censuses and Surveys
QI: Quality improvement
RCS: Royal College of Surgeons of England
ROC: Receiver operating characteristic (ROC) curve
SAH: Subarachnoid haemorrhage
SBNS: Society of British Neurological Surgeons
SCARF: Secondary Care Administrative Records Frailty
TARN: Trauma Audit and Research Network
TBI: Traumatic brain injury
