# Supplementary material for: Benchmarking short-term postoperative mortality across neurosurgery units: is hospital administrative data good enough for risk-adjustment?
Source: Acta Neurochir (Wien). 2023 May 27;165(7):1695–706. doi: 10.1007/s00701-023-05623-5 (PMC10319696; doi:10.1007/s00701-023-05623-5)

Benchmarking short-term postoperative mortality across neurosurgery units: is hospital administrative data good enough for risk-adjustment?

## Supplementary material

Journal: Acta Neurochirurgica

Authors: Adam J Wahba

Nick Phillips

Ryan K Mathew

Peter J Hutchinson

Adel Helmy

David A Cromwell

Corresponding author: Adam J Wahba

Clinical Effectiveness Unit, Royal College of Surgeons of England, 35-43 Lincoln's Inn Fields, London WC2A 3PE, UK

adam.wahba@nhs.net

## Index of captions for tables and figures

**Table S1**: List of procedures and OPCS codes in each procedure group

**Tables S2**: ICD-10 codes used in the RCS Charlson Comorbidity Index. The table denotes the codes for which the ‘look-back’ method was used to distinguish comorbidity from acute illness.

**Tables S3**: ICD-10 codes used in the Elixhauser Score. The table denotes the codes for which the ‘look-back’ method was used to distinguish comorbidity from acute illness, and the weighted points given to each disease category.

**Table S4:** Adjusted odds ratios (95% CI) from the final risk adjustment models for 30-day postoperative mortality for the overall cohort and the three subspecialty models.

**Table S5**: Characteristics of patients undergoing each procedure between April 2013 – March 2018 and 30-day mortality rates for the index procedures within the three subspecialties.

**Table S6**: Discriminatory ability of the best performing subspecialty risk-adjustment models when their performance was tested a key procedure within each subspecialty (compared to performance in non-key procedures). C-statistics are derived from the area under the ROC and the 95% CI is shown in parentheses.

**Figure S1 (a-d)**: Calibration plots showing the progressive change in calibration for models 1-5 in the overall cohort and the subspecialty models. E:O – calibration intercept, CITL – calibration in-the-large, AUC – area under the receiver operating characteristic curve (c-statistic).

**Figure S2**: Calibration plots of the best performing subspecialty risk-adjustment models when tested for a key procedure within each subspecialty (compared to the remaining procedures in the subspecialty). E:O – calibration intercept, CITL – calibration in-the-large, AUC – area under the receiver operating characteristic curve (c-statistic).

**Table S1:** List of procedures, OPCS codes and ICD-10 codes used to filter the procedures

| **Subspecialty** | **OPCS-4.8 Codes** | **Procedures** | **ICD-10 codes** |
| --- | --- | --- | --- |
| **Neuro-oncology surgery** | A012, A013, A021, A022, A023, A024, A025, A026, A028, A029, A041, A042, A043, A044, A045, A046, A048, A049, A081, A082, A083, A084, A085, A086, A088, A089, A106, A171, A181, B061, B068, B069, A381, A382, A383, A384, A385, A386, A388, A389, A431, A432, A438, A439 | Intracerebral tumour resection  Tumour biopsy  Resection of meningioma | C71x - Malignant neoplasm of brain, D33x - Benign neoplasm of brain and other parts of CNS, D43x - Neoplasm of uncertain or unknown behaviour of brain and CNS, C793 - Secondary malignant neoplasm of brain and cerebral meninges |
| **Key procedure – intracerebral tumour resection** | A012, A013, A021, A022, A023, A024, A025, A026 |  | C71x - Malignant neoplasm of brain, D33x - Benign neoplasm of brain and other parts of CNS, D43x - Neoplasm of uncertain or unknown behaviour of brain and CNS, C793 - Secondary malignant neoplasm of brain and cerebral meninges |
| **Neurovascular surgery** | L291, L292, L293, L294, L295, L296, L297, L298, L299, L301, L302, L303, L304, L305, L308, L309, L311, L313, L314, L318, L319, L331, L332, L333, L334, L338, L339, L341, L342, L343, L344, L348, L349, L351, L353, L358, L359, L371, L372, L373, L374, L378, L379, L381, L382, L383, L384 ,L388, L389, L391, L392, L393, L395, L398, L399, L751, L752, L753, L754, L755, L756, L758, L759, O011, O012, O013, O014, O018, O019, O021, O022, O023, O028, O029, O031, O032, O033, O038, O039, O041, O042, O048, O049, O051, O052, O053, O058, O059 | Open clipping of cerebral aneurysm  Transluminal treatment of cerebral aneurysm  Operations on dural arteriovenous fistula  Open treatment of AVM  Transluminal treatment of AVM | n/a |
| **Key procedure –**  **Clipping of cerebral aneurysm** | L331, L332, L333, L334, L338, L339 |  | n/a |
| **Trauma neurosurgery** | A072, A113, A203, A208, A209, A401, A408, A409, A411, V037, V053, V054, V057 | Removal of foreign body from tissue of brain  Evacuation of ASDH  Evacuation of EDH  Decompressive craniectomy  Monitoring of pressure in tissue of brain | S06x - Intracranial injury |
| **Key procedure – Evacuation of ASDH** | A411 |  | S06x - Intracranial injury |

**Tables S2:** ICD-10 codes used in the RCS Charlson Comorbidity Index. The table denotes the codes for which the ‘look-back’ method was used to distinguish comorbidity from acute illness.

| **Disease Category** | **ICD10 Codes** |
| --- | --- |
| Myocardial infarction | I21*, I22*, I23*, I252 |
| Congestive cardiac failure | I11, I13, I255, I42, I43, I50, I517 |
| Peripheral vascular disease | I70–I73, I770, I771, K551, K558, K559, R02, Z958, Z959 |
| Cerebrovascular disease | G45** - Transient cerebral ischaemic attacks and related syndromes (because G459 includes ‘spasm of cerebral artery’ >> SAH) |
|  | G46** - Vascular syndromes of brain in cerebrovascular diseases |
|  | I60** - Subarachnoid haemorrhage |
|  | I61** - Intracerebral haemorrhage |
|  | I62** - Other nontraumatic intracranial haemorrhage |
|  | I63** - Cerebral infarction |
|  | I64** - Stroke, not specified as haemorrhage or infarction |
|  | I65** - Occlusion and stenosis of precerebral arteries, not resulting in cerebral infarction |
|  | I66** - Occlusion and stenosis of cerebral arteries, not resulting in cerebral infarction |
|  | I67** - Other cerebrovascular diseases |
|  | I68** - Cerebrovascular disorders in diseases classified elsewhere |
|  | I69** - Sequelae of cerebrovascular disease |
| Dementia | A810, F00–F03, F051, G30, G31 |
| Chronic pulmonary disease | I26, I27, J40–J45, J46*, J47, J60–J67, J684, J701, J703 |
| Rheumatological disease | M05, M06, M09, M120, M315, M32–M36 |
| Liver disease | B18, I85, I864, I982, K70, K71, K721, K729, K76, R162, Z944 |
| Diabetes mellitus | E10–E14 |
| Hemiplegia or paraplegia | G114 |
|  | G81** - Hemiplegia |
|  | G82** - Paraplegia and tetraplegia |
|  | G83** - Other paralytic syndromes |
| Renal disease | I12, I13, N01, N03, N05, N07, N08, N171*, N172*, N18, N19*, N25, Z49, Z940, Z992 |
| Any malignancy | C00–C26, C30–C34, C37–C41, C43, C45–C58, C60–C76***, C80–C85, C88, C90–C97 |
| Metastatic solid tumour | C77–C79*** |
| AIDS/HIV infection | B20–B24 |

Notes:

| *Conditions defined as comorbidity only if present in an admission in the previous 12 months (Armitage 2010). |
| --- |
| **Additional codes, beyond those specified in Armitage et al 2010, defined as comorbidity only if present in an admission in the previous 12 months and not present in diag_01 . |
| ***C70-C72,C793,C794 excluded when the model was used to assess neuro-oncology procedures (except meningioma).  **Tables S3:** ICD-10 codes used in the Elixhauser Score, and the weighted points given to each disease category. |

| \| **Disease Category** \| **ICD10 Codes** \| **Points** \| \| --- \| --- \| --- \| \| Congestive heart failure \| I09.9, I11.0, I13.0, I13.2,I25.5, I42.0, I42.5–I42.9, I43.x, I50.x,P29.0 \| 7 \| \| Cardiac arrhythmias \| I44.1–I44.3, I45.6, I45.9,I47.x*, I48.x*, I49.x*, R00.0*,R00.1*, R00.8*, T82.1,Z45.0, Z95.0 \| 5 \| \| Valvular disease \| A52.0, I05.x–I08.x, I09.1,I09.8, I34.x–I39.x,Q23.0–Q23.3, Z95.2–Z95.4 \| -1 \| \| Pulmonary circulation disorders \| I26.x, I27.x, I28.0, I28.8,I28.9 \| 4 \| \| Peripheral vascular disorders \| I70.x, I71.x, I73.1, I73.8,I73.9, I77.1, I79.0,I79.2, K55.1, K55.8,K55.9, Z95.8, Z95.9 \| 2 \| \| Hypertension \| I10.x \| 0 \| \| Hypertension \| I11.x–I13.x, I15.x \| 0 \| \| Paralysis \| G04.1, G11.4, G80.1,G80.2, G81.x*, G82.x*,G83.0–G83.4*, G83.9* \| 7 \| \| Other neurological disorders \| G10.x–G13.x(excl G11.4 as in paralysis), G20.x–G22.x, G25.4, G25.5,G31.2, G31.8, G31.9,G32.x, G35.x–G37.x(G37.2*),G40.x*, G41.x*, G93.1*,G93.4, R47.0*, R56.x* \| 6 \| \| Chronic pulmonary disease \| I27.8, I27.9, J40.x–J47.x,J60.x–J67.x, J68.4,J70.1, J70.3 \| 3 \| \| Diabetes, uncomplicated \| E10.0, E10.1, E10.9,E11.0, E11.1, E11.9,E12.0, E12.1, E12.9,E13.0, E13.1, E13.9,E14.0, E14.1, E14.9 \| 0 \| \| Diabetes, complicated \| E10.2–E10.8,E11.2–E11.8, E12.2–E12.8, E13.2–E13.8,E14.2–E14.8 \| 0 \| \| Hypothyroidism \| E00.x–E03.x, E89.0 \| 0 \| \| Renal failure \| I12.0, I13.1, N18.x,N19.x*, N25.0, Z49.0–Z49.2, Z94.0, Z99.2 \| 5 \| \| Liver disease \| B18.X, I85.X, I86.4, I98.2, K70.X, K71.1, K71.3-K71.5, K71.7,K72.X-K74.X,K76.0,K76.2-K76.9,Z94.4 \| 11 \| \| Peptic ulcer disease excluding bleeding \| K25.7, K25.9, K26.7, K26.9,K27.7, K27.9, K28.7,K28.9 \| 0 \| \| AIDS/HIV \| B20.x–B22.x, B24.x \| 0 \| \| Lymphoma \| C81.x–C85.x, C88.x, C96.x,C90.0, C90.2 \| 9 \| \| Metastatic cancer \| C77.x–C80.x \| 12 \| \| Solid tumour without metastasis \| C00.x–C26.x, C30.x–C34.x,C37.x–C41.x, C43.x,C45.x–C58.x,C60.x–C76.x, C97.x \| 4 \| \| Rheumatoid arthritis / collagen vascular diseases \| L94.0, L94.1, L94.3, M05.x,M06.x, M08.x, M12.0,M12.3, M30.x, M31.0–M31.3, M32.x–M35.x,M45.x, M46.1, M46.8,M46.9 \| 0 \| \| Coagulopathy \| D65–D68.x, D69.1, D69.3–D69.6 \| 3 \| \| Obesity \| E66.x \| -4 \| \| Weight loss \| E40.x–E46.x, R63.4, R64 \| 6 \| \| Fluid & electrolyte disorders \| E22.2, E86.x*, E87.x* \| 5 \| \| Blood loss anaemia \| D50.0 \| -2 \| \| Deficiency anaemia \| D50.8, D50.9, D51.x–D53.x \| -2 \| \| Alcohol abuse \| F10, E52, G62.1, I42.6,K29.2, K70.0, K70.3,K70.9, T51.x, Z50.2,Z71.4, Z72.1 \| 0 \| \| Drug abuse \| F11.x–F16.x, F18.x, F19.x,Z71.5, Z72.2 \| -7 \| \| Psychoses \| F20.x (not F20.4 as it is in 'Depression'), F22.x–F25.x, F28.x,F29.x, F30.2, F31.2, F31.5 \| 0 \| \| Depression \| F20.4, F31.3–F31.5, F32.x,F33.x, F34.1, F41.2, F43.2 \| -3 \| |
| --- | --- | --- | --- | --- | --- | --- | --- | --- | --- | --- | --- | --- | --- | --- | --- | --- | --- | --- | --- | --- | --- | --- | --- | --- | --- | --- | --- | --- | --- | --- | --- | --- | --- | --- | --- | --- | --- | --- | --- | --- | --- | --- | --- | --- | --- | --- | --- | --- | --- | --- | --- | --- | --- | --- | --- | --- | --- | --- | --- | --- | --- | --- | --- | --- | --- | --- | --- | --- | --- | --- | --- | --- | --- | --- | --- | --- | --- | --- | --- | --- | --- | --- | --- | --- | --- | --- | --- | --- | --- | --- | --- | --- | --- | --- | --- | --- |

Notes: *Conditions defined as comorbidity only if present in an admission in the previous 12 months.

**Table S4:** Adjusted odds ratios (95% CI) from the final risk adjustment models for 30-day postoperative mortality for the overall cohort and the three subspecialty models.

|  | All Neurosurgery | Neuro-oncology surgery | Neurovascular surgery | Trauma neurosurgery |
| --- | --- | --- | --- | --- |
|  | Model 5: basic + deprivation + frailty + comorbidity (ES) | Model 5: basic + deprivation + frailty + comorbidity (ES) | Model 1: basic – (age, sex, admission type) | Model 3: basic + deprivation + frailty |
|  |  |  |  |  |
| Age | 1.00 (1.00, 1.01) | 1.03 (1.02, 1.03) | 1.02 (1.02, 1.03) | 0.99 (0.98, 1.00) |
| Male | 0.98 (0.88, 1.08) | 1.26 (1.09, 1.44) | 0.76 (0.63, 0.90) | 0.90 (0.81, 1.00) |
| Emergency admission | 3.84 (3.30, 4.47) | 2.61 (2.16, 3.16) | 10.7 (7.51, 15.28) | n/a |
| Subspecialty |  |  |  |  |
| Neuro-oncology surgery | 1 | n/a | n/a | n/a |
| Neurovascular surgery | 0.96 (0.83, 1.11) |  |  |  |
| Trauma neurosurgery | 1.72 (1.50, 1.98) |  |  |  |
| Socioeconomic deprivation |  |  |  |  |
| 1 (least) | 1 | 1 | n/a | 1 |
| 2 | 0.92 (0.84, 1.02) | 0.97 (0.72, 1.30) |  | 0.95 (0.82, 1.10) |
| 3 | 0.83 (0.73, 0.95) | 1.09 (0.87, 1.38) |  | 0.82 (0.66, 1.01) |
| 4 | 0.94 (0.87, 1.03) | 1.07 (0.88, 1.30) |  | 1.02 (0.88, 1.17) |
| 5 (Most) | 0.90 (0.81, 1.00) | 0.96 (0.75, 1.23) |  | 0.98 (0.83, 1.15) |
| Frailty |  |  |  |  |
| Fit | 1 | 1 | n/a | 1 |
| Mild | 1.27 (1.09, 1.49) | 1.49 (1.22, 1.82) |  | 0.95 (0.77, 1.18) |
| Moderate | 1.60 (1.37, 1.87) | 2.39 (1.89, 3.03) |  | 1.16 (0.93, 1.44) |
| Severe | 1.87 (1.61, 2.16) | 4.17 (3.12, 5.57) |  | 1.43 (1.18, 1.73) |
| Comorbidities (ES groups) |  |  |  |  |
| 1 | 1 | 1 | n/a | n/a |
| 2 | 1.46 (1.16, 1.83) | 1.14 (0.92, 1.41) |  |  |
| 3 | 1.25 (1.00, 1.54) | 1.44 (1.10, 1.88) |  |  |
| 4 | 1.19 (0.94, 1.52) | 1.42 (0.99, 2.02) |  |  |
| 5 | 1.60 (1.23, 2.10) | 1.84 (1.33, 2.56) |  |  |
| 6 | 2.06 (1.60, 2.65) | 1.47 (1.09, 1.96) |  |  |

**Table S5**: Characteristics of patients undergoing each procedure between April 2013 – March 2018 and 30-day mortality rates for the index procedures within the three subspecialties.

| **Intracerebral tumour resection** |  |  | **Clipping of cerebral aneurysm** |  |  |
| --- | --- | --- | --- | --- | --- |
| No. of patients | 13,524 |  | No. of patients | 1,950 |  |
| Mortality rate, n (%) | 330 | 2.4 | Mortality rate (%) | 97 | 5.0 |
| Average age, (median, IQR) | 57 (45 - 66) |  | Average age, (median, IQR) | 54 (47 - 63) |  |
| Male, n (%) | 7,405 | 54.8 | Male, n (%) | 596 | 30.6 |
| Emergency admission, n (%) | 3,788 | 28.0 | Emergency admission, n (%) | 1,114 | 57.1 |
| Comorbidities, n (%) |  |  | Comorbidities, n (%) |  |  |
| 0 | 7,161 | 53.0 | 0 | 892 | 45.7 |
| 1 | 3,449 | 25.5 | 1 | 677 | 34.7 |
| 2 | 1,980 | 14.6 | 2 | 292 | 15.0 |
| 3+ | 934 | 6.9 | 3+ | 89 | 4.6 |
| Frailty, n (%) |  |  | Frailty, n (%) |  |  |
| Fit | 5,513 | 40.8 | Fit | 550 | 28.2 |
| Mild | 4,657 | 34.4 | Mild | 733 | 37.6 |
| Moderate | 2,709 | 20.0 | Moderate | 526 | 27.0 |
| Severe | 645 | 4.8 | Severe | 141 | 7.2 |
|  |  |  |  |  |  |
| **Evacuation of ASDH** |  |  |  |  |  |
| No. of patients | 6,737 |  |  |  |  |
| Mortality rate (%) | 613 | 9.1 |  |  |  |
| Average age, (median, IQR) | 75 (61 - 83) |  |  |  |  |
| Male, n (%) | 4,672 | 69.3 |  |  |  |
| Emergency admission, n (%) | 6,737 | 100 |  |  |  |
| Comorbidities, n (%) |  |  |  |  |  |
| 0 | 2,771 | 41.1 |  |  |  |
| 1 | 1,882 | 27.9 |  |  |  |
| 2 | 1,186 | 17.6 |  |  |  |
| 3+ | 898 | 13.3 |  |  |  |
| Frailty, n (%) |  |  |  |  |  |
| Fit | 725 | 10.8 |  |  |  |
| Mild | 1,524 | 22.6 |  |  |  |
| Moderate | 2,241 | 33.3 |  |  |  |
| Severe | 2,247 | 33.4 |  |  |  |

**Table S6**: Discriminatory ability of the best performing subspecialty risk-adjustment models when their performance was tested a key procedure within each subspecialty (compared to performance in non-key procedures). C-statistics are derived from the area under the ROC and the 95% CI is shown in parentheses.

|  | C-statistic |
| --- | --- |
| Neuro-oncology surgery (Model 5) |  |
| Intracerebral tumour resection | 0.725 (0.698 - 0.753) |
| Other neuro-oncology procedures | 0.757 (0.732 - 0.781) |
| Neurovascular surgery (Model 1) |  |
| Clipping of cerebral aneurysm | 0.769 (0.732 - 0.806) |
| Other neurovascular procedures | 0.721 (0.703 - 0.740) |
| Trauma neurosurgery (Model 3) |  |
| Evacuation of ASDH | 0.610 (0.588 - 0.633) |
| Other neurosurgical trauma procedures | 0.673 (0.647 - 0.700) |

**Figure S1 (a-d)**: Calibration plots showing the progressive change in calibration for models 1-5 in the overall cohort and the subspecialty models.


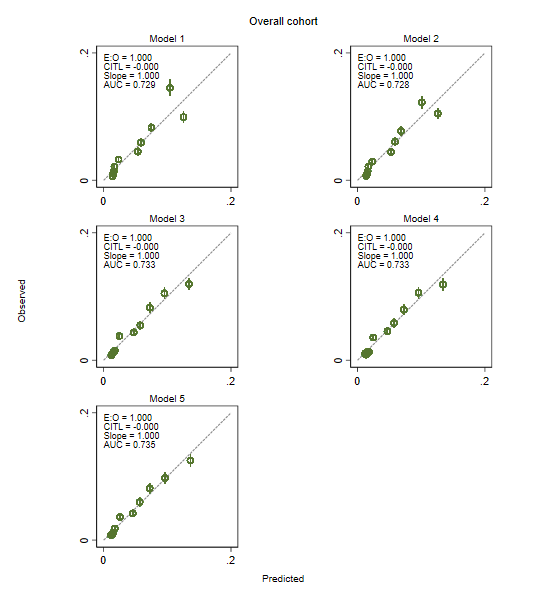


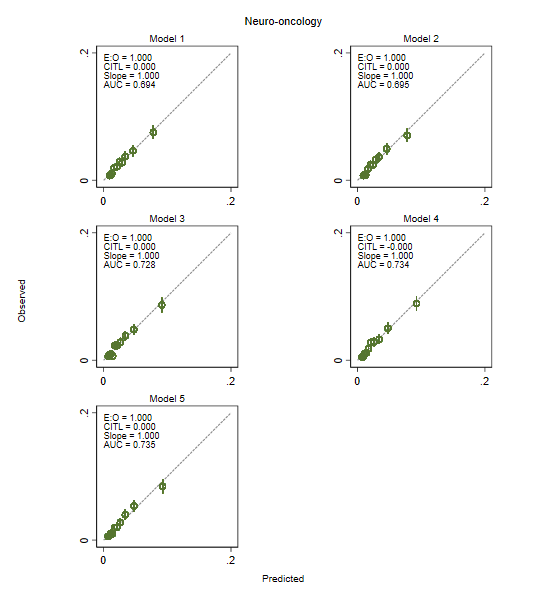


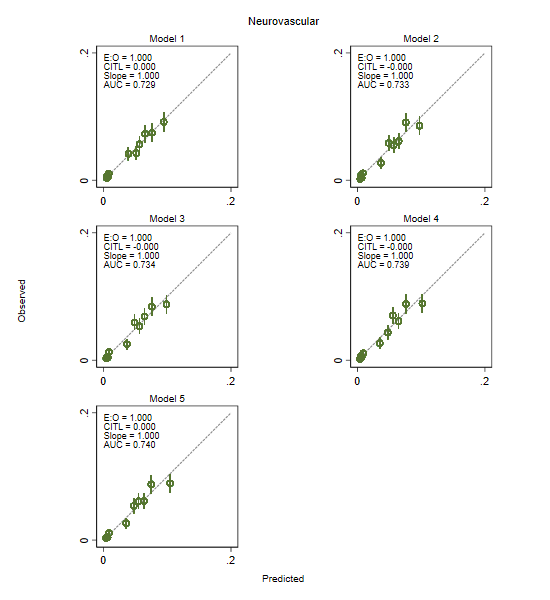

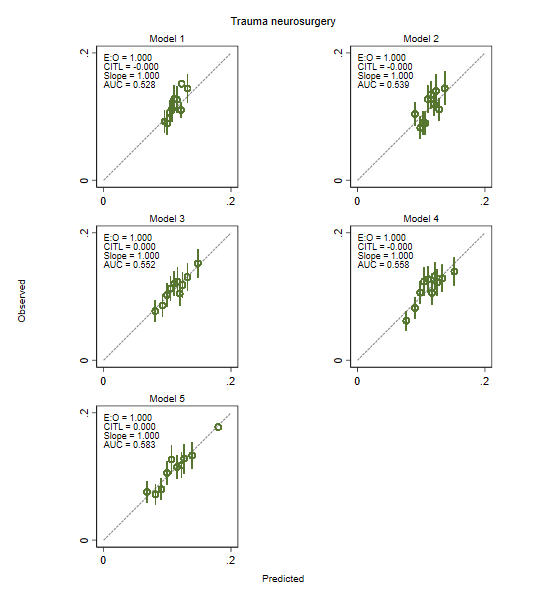


**Figure S2**: Calibration plots of the best performing subspecialty risk-adjustment models when tested for a key procedure within each subspecialty (compared to the remaining procedures in the subspecialty).


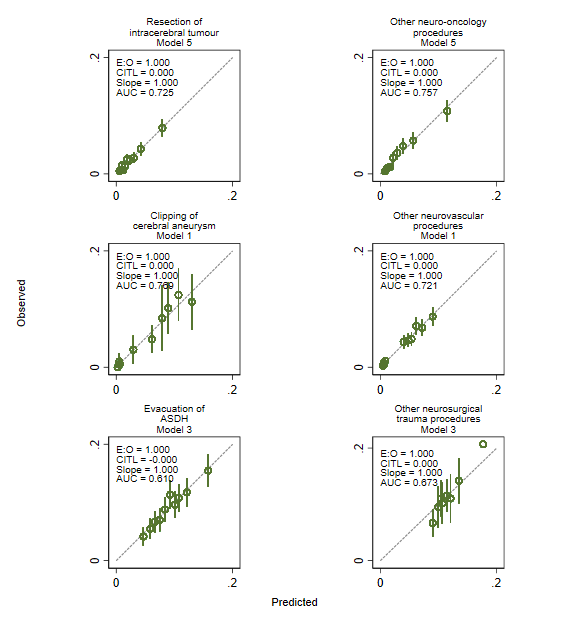

Supplement: Supplementary file 1 — (DOCX 214 kb) [file 701_2023_5623_MOESM1_ESM.docx]
